# Scraps

**Published:** 1896-06

**Authors:** 


### SCRAPS

PICKED UP BY THE ASSISTANT-EDITOR.

---

**Clinical Records** (15).—A groove in a hole is indeed a curiosity. An item in one of the newspapers recounts the sad case of a Staten Island dentist who broke a tooth that he was extracting, and was struck in the eye by a fragment of it. "It was discovered," says the article, "that a groove had been made in the pupil of the eye by the flying particle of tooth." It is added that the doctor in attendance has "but few hopes" of saving the sight of the injured eye.—*New York Medical Journal.*

**"The Weary Treadmill."**—Dr. H. O. Walker, in his Presidential Address before the Michigan State Medical Society, urged the importance of the yearly holiday to relieve the strain described in the following lines:—

To be day and night at the beck and call
  Of men who cheat, and women who lie,
To know how often the scoundrels live,
  And see with sorrow the dear ones die;
To be laughed to scorn as the man who fails
  When nature claims her terrible debt,
To give a mother her firstborn's smile
  And leave the eyes of the husband wet;
To face and brave the gossip and stuff
  That travels about through a country town,
To be thrown in the way of hysterical girls
  And live all terrible scandals down,
To study at night—in papers hear
  Of new diseases and human ills,
To work like a slave for weary years
  And then be cursed when you send your bills.

**A Smallpox Visitation.**—The tremendous destruction of life caused by smallpox, previous to the discovery of vaccination, can hardly be realised in our day. The disease was never absent from the chief towns, the victims in London numbering thousands yearly; but it occasionally became epidemic, and was as destructive as was the plague in the seventeenth century or the cholera within living memory. A frightful outbreak occurred in 1758, when one-tenth of the total mortality was due to it. Gloucestershire especially suffered severely, and in Cirencester the havoc was so great that the neighbouring farmers refused to enter the town, and the markets were held in the outskirts. At length, after the scourge had raged for over three months, the following advertisement, signed by the churchwardens, overseers, and high constables, was published in the *Gloucester Journal*:—"Cirencester, June 27, 1758. Whereas the smallpox is greatly upon the decline in this town, and must in a short time be entirely over, *there being but few people remaining to have it*, this is to give notice that whoever shall presume to bring any person or persons from any distant place or places into this town (not belonging to this parish) having the smallpox, or in order to have it here, shall be prosecuted with the utmost rigour of the law." Similar notices were published by the authorities of Tewkesbury, Winchcombe, and other towns.—N.R. in *Bristol Mercury*.

**Medical Philology** (XVIII).—The *Promptorium* (1440) gives "CODDE, of mannys pryuyte. *Piga, mentula.*" Pynson's printed edition reads "preuy membris" and "*testiculus, fiscus.*" The *New English Dictionary* says that Cod was improperly used in the plural to signify the testicles.[1] In this way it served to form the point of one of the dubious pleasantries which occurred in the course of Touchstone's wooing of Jane Smile (*As You Like It*, II. iv. 51-4). But the *Promptorium* entry, although not very definite in its Latin equivalents, shows that with this signification it was also used in the singular number. This is also shown in Cotgrave, *s.v.* "couille," "couillon," etc.

Early medical books also used "cods" to signify "testicles." Phaer's *Regiment of Life* (1553), under the heading of "Swelling of the coddes," describes many "a goodly plaister for swellyng of the stones" (sig. T. 4 *verso* and sig. Aa 5 *verso*). One of the sections in Nicholas Udall's translation (1559) of the *Treatyse of Anatomie* by Geminus, is entitled: "Of the purce conteynynge the Testicles called commonly the Coddes" (sig. A. 5 *recto*). Most anatomical and surgical works of a somewhat later period restricted the use of the term

[1] "Balocke cod" was Middle English for testicle. In the *Journal* for Sept., 1890, in a note on "the familiar name by which the schoolboy calls his testicles" I referred to this with some detail.

to either the scrotum or its covering, although occasionally in such the plural of the word is used for the testicles (see Mosan's translation of Wirsung's *General Practise of Physicke*, 1617, p. 276; Levens's *Path-way to Health*, 1632, p. 49 *verso*; Culpeper's *Last Legacy*, "Febrilia," 1656, p. 50). Helkiah Crooke, in his *Body of Man* (1615), says "the cod is a rugous and thin skin" (p. 250). Culpeper, in his translation (1677) of *The Anatomy of the Body of Man* by Veslingus, says of the scrotum, "We in English call it the Cods" (p. 24). Gibson's *Anatomy of Humane Bodies Epitomized* (4th Ed., 1694, p. 147) describes the testicles as hanging "at the root of the Yard, in the Cod." "A New Chirurgical Dictionary" added to Le Dran's *Observations in Surgery* defines "Pneumatocele" as "a Wind-Rupture in the Scrotum, or Cod" (3rd Ed., Tr. by J. S., 1758); and Percival Pott, in his *Chirurgical Works* (1783), says that ruptures "are called *inguinal*, *scrotal*, *femoral*, *umbilical*, and *ventral*, as they happen to make their appearance in the groin, cod, thigh, navel, or belly" (vol. ii., p. 14). All the books mentioned in this paragraph can be seen in the Bristol Medical Library.

Cod was used to denote a variety of things connected with its etymological origin of bag or pillow. Among other things it signified a purse, a term often now applied to the scrotum; and peascod is still familiar.

One of the most interesting points in connection with the word is the suggestion (see Skeat, *Etymological Dictionary*) that "Coddle" in the sense of "to make effeminate" literally means "to castrate," and is made up of *cod* with the weakening suffix *-le*, which forms part of such words as article, cradle, molecule, nucleus, particle. Skeat thinks that the passage in Beaumont and Fletcher's *Philaster* (1611) bears this meaning, where the Captain threatening Pharamond says:

> "Down with your noble blood; or, as I live,
> I 'll have you coddled." (v. iv. 30–1.)

In the otherwise tight breeches worn about the period of the sixteenth century there was, for the accommodation of the genital organs, a special arrangement known as the "Cod-piece," which was ornamented [1] and tied up with "points" of lace or silk. The literature of the time abounds in references to this conspicuous portion of attire, and we need go no further than Shakspere for sufficient instances. When Julia, in man's dress, was about to seek Proteus, her maid insisted upon the assumed breeches being provided with a cod-piece (*Two Gentlemen of Verona*, II. vii. 55). Borachio called attention to the anachronism in art which the tapestry-makers committed in representing Hercules with a cod-piece (*Much Ado About Nothing*, III. iii. 146). Not only was it conspicuous, but it was also made to serve useful purposes. Lucetta's acquaintance with the fashions enabled her to inform Julia that the only correct form was that which allowed it to be used as a pincushion. It was sometimes provided with a pocket. One of the accomplishments of Autolycus was his ability to "geld a cod-piece of a purse" (*Winter's Tale*, IV. iv. 623).

A piece of folk-lore which gathered about this important site finds expression in the French *esguillette nouée*, which Cotgrave (1611) renders "the charming of a man's codpeece-point so, as hee shall not be able to use his owne wife, or woman (though he may use any other)." Cotgrave adds "this impotencie is supposed to come by the force of certaine words vttered by the charmer, while he ties a knot on the parties codpeece-point."

In metaphorical language cod-piece did duty for its contents. When that man about town, Lucio, learned that his friend Claudio was condemned to death for having got Juliet with child, he thought it a ruthless thing that Angelo should have revived an obsolete Viennese law to inflict such a punishment merely for "the rebellion of a cod-piece" (*Measure for Measure*, III. ii. 122). Biron apostrophises Cupid as "king of cod-pieces" (*Love's Labour 's Lost*, III. 186); and allusion to it by the Fool will be found in *Lear*, III. ii. 27, 40.

Women wore similar appendages to receive the breasts. Harrison (1577), who was alarmed at the little difference between the dress of the two sexes, and who said that it "passed his skill to discern whether they were men or women," speaks of women's "doublets with pendant codpeeses on the brest full of iags & cuts" (*Description of England*, New Shakspere Soc. ed., 1877, Part I., p. 170).

1 The stranger in "Slawkenbergius' Tale" in *Tristram Shandy*, when he arrived at Strasburg "put his breeches with his fringed cod-piece on."

